# Timing of Antibiotics in ICU Pneumonia: An Observational Association Between Early Treatment and Higher Mortality

**DOI:** 10.3390/antibiotics15010049

**Published:** 2026-01-03

**Authors:** Josef Yayan, Kurt Rasche

**Affiliations:** Department of Internal Medicine, Division of Pulmonary, Allergy and Sleep Medicine, HELIOS Clinic Wuppertal, Witten/Herdecke University, Heusnerstr. 40, 42283 Wuppertal, Germany

**Keywords:** pneumonia, ICU, antibiotic timing, mortality, antimicrobial stewardship

## Abstract

**Background:** Early administration of antibiotics is commonly recommended for pneumonia in intensive care unit (ICU) patients. However, the clinical benefit of very early empirical treatment remains uncertain and may reflect differences in illness severity, baseline risk, or care pathways, particularly in non-septic or hemodynamically stable ICU populations. **Methods:** We performed a retrospective cohort study using the Medical Information Mart for Intensive Care IV (v2.2) database to evaluate the observational association between antibiotic timing and in-hospital mortality among adult ICU patients with pneumonia. Patients were categorized as receiving early (<3 h) or delayed (≥3 h) antibiotic therapy after ICU admission. A multivariable logistic regression model adjusted only for age and sex. Given the absence of detailed severity-of-illness measures, no causal inference was intended, and all analyses were considered hypothesis-generating. Additional analyses exploring antibiotic class, dosing frequency, and combination therapy were conducted in an exploratory manner, given substantial variation in sample sizes and a high risk of confounding by indication, misclassification, immortal-time, and survivorship bias. **Results:** Among 7569 ICU patients with pneumonia, 56.5% received antibiotics within three hours of ICU admission. Early antibiotic initiation was associated with higher in-hospital mortality than delayed therapy (26.1% vs. 21.5%; OR 1.30, 95% CI 1.16–1.44; *p* < 0.001). Because validated severity-of-illness measures were unavailable, residual confounding and confounding by indication are likely and may largely explain this association. A potential signal of increased mortality was observed in patients receiving ≥3 doses of levofloxacin (OR 4.39, 95% CI 1.13–17.02); however, this subgroup was small and the finding is highly susceptible to survivorship and indication bias. Mortality appeared lower in patients receiving two or three antibiotics compared with monotherapy, but marked group imbalances, lack of restriction or stratification, and clinical selection effects limit interpretability. Regimens involving ≥4 agents were rare and primarily associated with prolonged ICU length of stay rather than a clear mortality difference. **Conclusions:** In this large retrospective ICU cohort, very early antibiotic administration for pneumonia was observationally associated with higher in-hospital mortality. Causality cannot be inferred, and early treatment likely represents a marker of higher baseline risk or clinical urgency rather than a harmful exposure. These findings challenge the assumption that earlier antibiotic initiation is uniformly beneficial and underscore the importance of individualized, stewardship-aligned, and context-dependent decision-making regarding antimicrobial timing and intensity in critically ill patients.

## 1. Introduction

Pneumonia is one of the most frequent reasons for intensive care unit (ICU) admission and remains associated with substantial morbidity and mortality worldwide [1,2]. Early and appropriate antibiotic therapy is traditionally considered fundamental to the management of suspected bacterial pneumonia, and clinical guidelines commonly recommend initiating treatment within one to three hours of diagnosis to improve outcomes [3,4,5]. However, these recommendations are largely derived from studies of septic shock or emergency department populations and may not be directly applicable to all ICU pneumonia patients, particularly those without shock or clear sepsis at presentation.

Whether such time-sensitive treatment strategies translate into improved survival for ICU patients whose primary diagnosis is pneumonia—particularly those without overt sepsis, shock, or hemodynamic instability—remains uncertain [6,7]. Observational studies examining the relationship between antibiotic timing and outcomes in sepsis have produced inconsistent and sometimes conflicting results. While some analyses report higher mortality with delayed therapy [8,9], others have found no consistent benefit from very early antibiotic administration and, in some cases, have even suggested an observed association with worse outcomes [10,11]. Importantly, many of these investigations evaluated heterogeneous ICU cohorts or all-cause sepsis populations, limiting their applicability to pneumonia-specific clinical presentations and raising concern for confounding by indication and time-related bias [12].

In addition to the timing of antibiotic initiation, several dimensions of antimicrobial therapy may influence outcomes in critically ill patients, including agent selection, dosing intensity, and the use of combination regimens. Broad empirical therapy is frequently employed to ensure adequate initial coverage, yet inappropriate selection or excessive combinations may contribute to antimicrobial resistance, adverse drug reactions, and treatment failure [13,14]. Moreover, very early empirical treatment may obscure diagnostic clarification, complicate microbiological interpretation, and influence downstream therapeutic decisions. These competing risks highlight the tension between timely empirical therapy and antimicrobial stewardship in the ICU, underscoring the challenge of establishing universally applicable therapeutic strategies for ICU pneumonia.

Given these uncertainties, we sought to evaluate the observational association between antibiotic timing and in-hospital mortality in adult ICU patients with pneumonia using a large, real-world dataset. We additionally examined patterns of antibiotic class, dosing frequency, and combination therapy to better understand contemporary treatment practices. Our aim was explicitly not to infer causality, but rather to reassess the commonly held assumption that “earlier is always better” and to generate hypotheses that may inform a more individualized, risk-aware and stewardship-aligned approach to antimicrobial decision-making in the ICU setting.

## 2. Results

**Figure 1:**
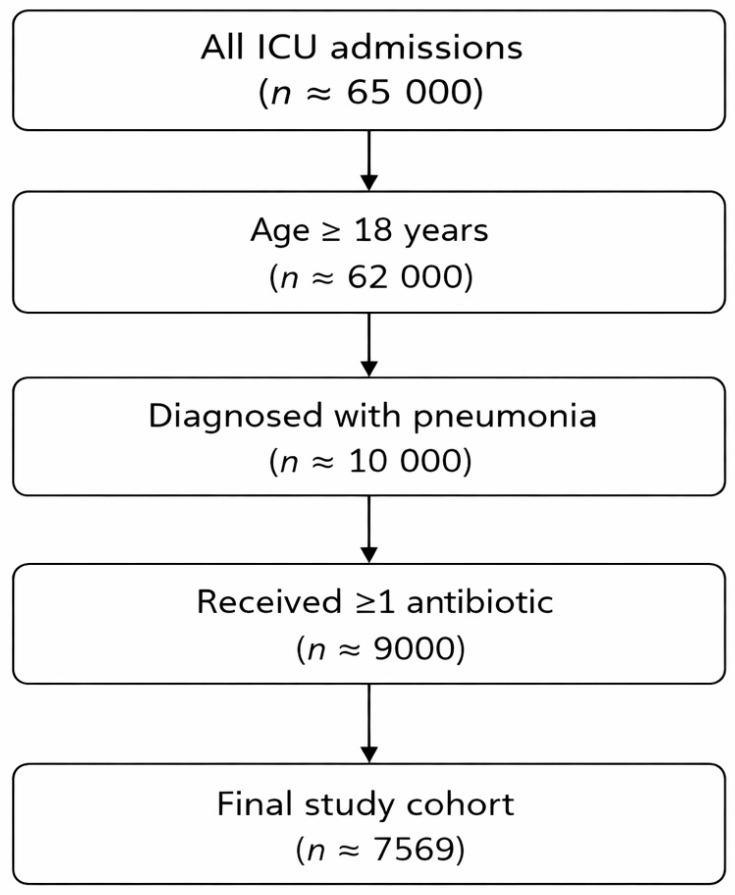
Flowchart of patient inclusion. Flowchart illustrating the selection of the final study cohort from the Medical Information Mart for Intensive Care IV (MIMIC-IV) database. After applying age eligibility (≥18 years), diagnosis-based case identification, documentation of at least one intensive care unit (ICU)-recorded antibiotic administration, and data completeness criteria, the final analytic cohort comprised 7569 patients admitted to the ICU with pneumonia. Because cohort assembly relied on administrative coding and electronic timestamps, diagnostic misclassification and selection bias cannot be fully excluded.

A total of 7569 adult ICU patients with pneumonia met the inclusion criteria (Figure 1). Of these, 4279 patients (56.5%) received their first antibiotic dose within ≤3 h of ICU admission (early group), whereas 3290 patients (43.5%) received antibiotics >3 h after admission (delayed group). Baseline characteristics, including mean age (63.6 years in both groups) and sex distribution (57% male overall), were similar between groups (Table 1 and Table 2); however, no validated measures of illness severity were available, and residual baseline differences related to clinical acuity cannot be excluded.

### 2.1. Mortality and Length of Stay

Patients in the early antibiotic group exhibited a higher crude in-hospital mortality rate compared with those in the delayed group (26.1% vs. 21.5%). Median ICU length of stay (LOS) was slightly shorter among early-treated patients (3.4 vs. 4.0 days), whereas mean ICU LOS was longer in the delayed group (7.4 ± 9.2 vs. 6.7 ± 8.7 days), consistent with the right-skewed LOS distribution typical of ICU populations (Table 1). Importantly, ICU LOS is a post-admission, downstream variable that is strongly influenced by survival status, discharge practices, and clinical decision-making over time. Because ICU LOS is influenced by survivorship, discharge practices, and downstream clinical trajectories, these differences should be interpreted strictly descriptively rather than causally and should not be considered evidence of baseline group comparability or treatment effect.

### 2.2. Antibiotic Use Patterns

The most frequently prescribed antibiotics were cefepime, ceftriaxone, piperacillin–tazobactam, azithromycin, cefazolin, levofloxacin, amoxicillin–clavulanic acid, and ceftazidime. Because many patients received more than one agent, the total number of antibiotic prescriptions exceeded the number of treated individuals (Table 3). These frequencies reflect observed prescribing patterns and do not imply comparability between treatment groups or causal associations with outcomes.

### 2.3. Multivariable Analysis

In a multivariable logistic regression model adjusting only for age and sex, early antibiotic initiation remained associated with higher in-hospital mortality (OR 1.30, 95% CI 1.16–1.44; *p* < 0.0001). Increasing age was also associated with higher mortality (OR per year 1.02, 95% CI 1.02–1.02; *p* < 0.0001), whereas sex was not a significant predictor (Table 4). Additional covariates shown in Table 4, including ICU length of stay, represent downstream or post-exposure variables and were included solely for exploratory, non-etiologic purposes; these terms should not be interpreted as baseline confounder adjustment. Because validated severity-of-illness scores and physiologic markers were unavailable, residual confounding—particularly confounding by indication and time-related bias—cannot be excluded, and these estimates should not be interpreted causally.

### 2.4. Antibiotic-Specific Associations

When individual antibiotic agents were evaluated, heterogeneous mortality patterns were observed across drug classes (Table 5). However, interpretation of these comparisons is limited for several reasons:

1. Substantial variation in group sizes, including small samples for several antibiotics;
2. Reported *p* values pertain to ICU LOS rather than mortality, precluding formal inference regarding agent-specific mortality differences;
3. Antibiotic selection likely reflects underlying illness severity, suspected pathogens, prior or pre-ICU antibiotic exposure, or previous treatment decisions, introducing strong indication bias that could not be reduced through restriction, stratification, or sensitivity analyses.

Observed mortality also varied by the number of antibiotics administered. Patients receiving monotherapy had a higher crude mortality rate (24.7%) than those treated with two (10.0%) or three antibiotics (10.5%). However, these combination-therapy groups were small and likely represent clinically selected subpopulations, such as patients with confirmed infections or identifiable pathogens who survived long enough to receive multiple agents. Therefore, these findings should be regarded as purely descriptive and hypothesis-generating, with a high risk of survivorship and indication bias. The very small group receiving ≥4 antibiotics (*n* = 3) experienced no in-hospital deaths but had prolonged ICU LOS (Table 6); the extremely limited sample size and survivorship-dependent exposure definition preclude meaningful interpretation.

### 2.5. Dosing Frequency Analysis

In exploratory univariable logistic regression, administration of ≥3 doses of levofloxacin was associated with higher in-hospital mortality (OR 4.39, 95% CI 1.13–17.02; *p* = 0.033). Given the small subgroup size and the inherent survivorship and indication bias (patients must survive long enough to receive multiple doses, and no restriction, stratification, or sensitivity analyses were performed to mitigate this bias), this finding should be interpreted with extreme caution and considered purely hypothesis-generating only. No other antibiotics demonstrated statistically significant associations between dosing frequency and mortality (Table 7).

### 2.6. Visualization of Dose-Related Effects

**Figure 2:**
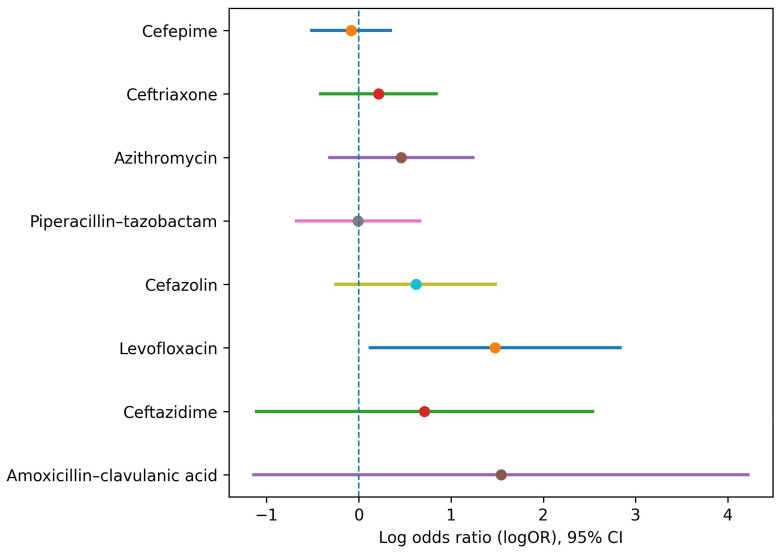
Coefficient plot of antibiotic-specific associations with in-hospital mortality (≥3 vs. <3 doses). This coefficient plot displays the estimated log odds ratios (logORs) with 95% confidence intervals (CIs) for in-hospital mortality associated with receiving ≥3 versus <3 doses of the eight most frequently prescribed antibiotics in patients admitted to the intensive care unit (ICU). All estimates are derived from univariable analyses and represent downstream exposure. Because patients must survive long enough to receive multiple doses, the displayed associations are subject to substantial survivorship and indication bias and should be interpreted as exploratory and hypothesis-generating only. The vertical dashed line at logOR = 0 represents no association (odds ratio (OR) = 1). Points to the right of the line indicate higher odds of mortality, whereas points to the left indicate lower odds of mortality.

A coefficient plot summarizing the odds ratios for in-hospital mortality associated with receiving ≥3 versus <3 doses of commonly prescribed antibiotics is shown in Figure 2. Cefpodoxime proxetil (*n* = 12) was excluded due to insufficient sample size to generate stable effect estimates. All estimates are derived from univariable analyses and reflect downstream exposure; because patients must survive long enough to receive multiple doses, the displayed associations are subject to substantial survivorship and indication bias and should be interpreted as exploratory and hypothesis-generating only.

## 3. Discussion

In this large retrospective cohort of ICU patients with pneumonia, early antibiotic administration—defined as treatment within three hours of ICU admission—was associated with higher in-hospital mortality compared with delayed administration. Although this finding contrasts with traditional sepsis management guidelines that emphasize rapid empirical treatment [4,5,7], it is consistent with emerging observational evidence suggesting that very early antibiotic initiation does not uniformly translate into improved outcomes, particularly in patients without septic shock or profound hemodynamic instability [15,16].

A central challenge in interpreting this association is the high likelihood of confounding by indication. Patients receiving antibiotics earlier may have appeared more acutely ill, prompting expedited treatment based on clinical urgency rather than anticipated therapeutic benefit [17]. Because validated severity-of-illness measures such as SOFA or APACHE II scores were unavailable, residual confounding is expected and may partially or fully explain the observed mortality differences [18,19]. Accordingly, the observed association between very early antibiotic therapy and higher mortality should not be interpreted as causal.

Another possible explanation is that modest delays in antimicrobial initiation may allow clinicians additional time to refine diagnostic assessment. Initiating empirical therapy very early may obscure microbiological results, reduce the accuracy of pathogen identification, and complicate selection of targeted therapy. Moreover, premature broad-spectrum exposure may contribute to antimicrobial resistance, disrupt the microbiome, or increase the risk of complications such as *Clostridioides difficile* infection [20,21]. Early empirical treatment may also impede subsequent diagnostic precision, influencing downstream therapeutic decisions [22]. While these mechanisms are biologically plausible, the present data do not permit definitive causal inference.

Findings related to antibiotic selection and dosing must be interpreted with caution. The observation that patients receiving ≥3 doses of levofloxacin exhibited higher mortality arose from a small and clinically selected subgroup, increasing the likelihood of chance findings, survivorship bias, or indication bias. Known adverse effects of fluoroquinolones—such as QT prolongation, neurotoxicity, or dysglycemia—may disproportionately affect critically ill patients [23]; however, the present analysis is insufficient to support agent-specific safety or efficacy conclusions. These findings should therefore be regarded as exploratory and hypothesis-generating.

Similarly, the lower observed mortality among patients receiving two or three antibiotics compared with monotherapy must be interpreted within the context of marked group-size imbalances and likely clinical selection effects. Combination therapy may reflect clearer infection identification, microbiological confirmation, or more aggressive management in relatively stable patients rather than an intrinsic benefit of multi-agent regimens. The extremely small number of individuals receiving four or more antibiotics precludes any meaningful interpretation of outcomes or potential toxicity.

Overall, these findings suggest that the timing, selection, and intensity of antimicrobial therapy in ICU pneumonia require individualized clinical judgment rather than universal acceleration. Strict adherence to a “sooner is always better” paradigm may not be appropriate in all clinical contexts and may be counterproductive when diagnostic uncertainty is high [24]. Future investigations—ideally prospective studies incorporating detailed physiologic severity metrics, microbiological data, and time-varying analytic approaches—are needed to determine the optimal timing and strategy for antibiotic therapy in critically ill patients with pneumonia.

## 4. Limitations

This study has several important limitations. First, as a retrospective observational analysis, it is inherently subject to confounding, selection bias, and information bias. Although the primary regression model adjusted for age and sex, adjustment for illness severity was not possible because validated clinical measures such as SOFA or APACHE II scores, lactate levels, shock status, ventilator use, and comorbidity indices were unavailable. This limitation substantially restricts the ability to disentangle the effect of antibiotic timing from underlying differences in clinical acuity. Second, antibiotic timing was derived from electronic medication timestamps rather than direct bedside administration records, introducing potential exposure misclassification. In addition, antibiotic use prior to ICU admission could not be reliably captured, limiting the ability to distinguish new initiation from continuation of pre-ICU therapy. Third, the exposure definitions used in several analyses introduce time-related bias. Patients in delayed or higher-dose categories necessarily survived long enough to receive antibiotics or multiple doses, resulting in immortal-time and survivorship bias that could not be fully addressed in the absence of time-varying or landmark analyses. No restriction, stratification, or sensitivity analyses were performed to mitigate this bias. Fourth, microbiological etiology and the appropriateness of empirical antibiotic selection were not assessed because culture data were not consistently linkable to antibiotic administration records. Consequently, the relationship between timing, appropriateness of therapy, and outcomes could not be evaluated. Fifth, secondary analyses examining antibiotic number and dosing frequency were exploratory and involved small, highly selected subgroups. Observed patterns—such as lower mortality among patients receiving multiple antibiotics—are therefore more likely to reflect indication bias or clinical selection rather than true therapeutic effects. Adverse drug reactions, antimicrobial resistance, and long-term outcomes were not evaluated. Finally, this study was conducted at a single academic medical center in the United States, potentially limiting generalizability to ICUs with different patient populations, resistance patterns, and antimicrobial stewardship practices. Despite these limitations, the study provides valuable real-world, hypothesis-generating insights into antibiotic timing and treatment patterns among critically ill patients with pneumonia and highlights the need for rigorously designed prospective investigations.

## 5. Material and Methods

### 5.1. Data Source

This retrospective cohort study was conducted using the Medical Information Mart for Intensive Care IV (MIMIC-IV), version 2.2. MIMIC-IV contains de-identified clinical data from more than 70,000 ICU admissions at Beth Israel Deaconess Medical Center in Boston, Massachusetts, collected between 2008 and 2019. To protect patient privacy, all calendar dates are randomly shifted; however, intra-patient time intervals remain valid, permitting temporal analyses. The database includes detailed demographic information, medication administration records, laboratory values, and clinical documentation.

### 5.2. Study Population

Adult patients (≥18 years) admitted to any ICU with a diagnosis of pneumonia were eligible for inclusion. Pneumonia was identified using ICD-9 and ICD-10 diagnostic codes and may have included community-acquired, hospital-acquired, or aspiration pneumonia. Patients were required to have received at least one recorded antibiotic dose during their ICU stay. Individuals were excluded if the timing of antibiotic administration or ICU admission was missing. Because case identification relied on administrative coding, diagnostic misclassification cannot be excluded.

### 5.3. Exposure Definition

The exposure of interest was the time interval between ICU admission and administration of the first ICU-recorded antibiotic dose. Patients were categorized into two groups:

- Early administration: <3 h after ICU admission
- Delayed administration: ≥3 h after ICU admission

Timing was derived from the variables *abx_starttime* and *icu_intime*. Because bedside administration may not perfectly correspond to electronic timestamps, exposure misclassification is possible. In addition, patients in the delayed group necessarily survived long enough to receive antibiotics, introducing the potential for immortal-time bias, which could not be fully addressed in the present analysis. Antibiotic exposure prior to ICU admission could not be reliably captured; therefore, timing reflects ICU-recorded administration and may not represent true initiation of therapy.

### 5.4. Outcomes

The primary outcome was all-cause in-hospital mortality.

Secondary outcomes included:

- ICU length of stay (LOS)
- Exploratory associations between antibiotic class and mortality
- Exploratory associations between dosing frequency (≥3 vs. <3 doses) and mortality
- Exploratory associations between the number of antibiotics administered and mortality

All secondary analyses were prespecified as exploratory, given the limited availability of severity-of-illness measures and substantial heterogeneity in antibiotic use patterns.

### 5.5. Covariates

Covariates included age, sex, and ICU type. Binary variables were generated for antibiotic timing, dosing frequency, and antibiotic class. Key clinical severity indicators—including SOFA or APACHE II scores, lactate levels, shock status, ventilator use, and comorbidity burden—were unavailable, and therefore residual confounding was anticipated.

### 5.6. Statistical Analysis

Descriptive statistics were used to summarize patient characteristics. Continuous variables (e.g., age, ICU LOS) were presented as means with standard deviations or medians with ranges, depending on distribution. Categorical variables (e.g., sex, mortality) were summarized as counts and percentages.

Group differences were assessed using:

- Chi-square tests for categorical variables
- Independent *t*-tests or Mann–Whitney U tests for continuous variables, as appropriate

To evaluate the association between antibiotic timing and in-hospital mortality, a multivariable logistic regression model was constructed adjusting for age and sex only. Because severity-of-illness measures were unavailable, the model was not intended to support causal inference.

Exploratory univariable logistic regression analyses were performed to assess associations between dosing frequency (≥3 vs. <3 doses) and mortality for commonly prescribed antibiotics. Given the small size of several subgroups and the risk of indication and survivorship bias, these analyses were interpreted cautiously. Exact *p* values were calculated using VassarStats: Website for Statistical Computation (R. Lowry; http://vassarstats.net; accessed on 15 September 2025) when event counts were sparse.

All analyses were conducted for hypothesis-generating purposes, and no corrections for multiple comparisons were applied.

## 6. Conclusions

In this large retrospective cohort of ICU patients with pneumonia, early antibiotic administration—defined as within three hours of ICU admission—was observationally associated with increased in-hospital mortality. Because of the observational study design and limited adjustment for illness severity, causality cannot be inferred, and early treatment likely reflects differences in baseline risk, clinical urgency, or care pathways rather than a direct harmful effect. Nevertheless, these findings challenge the assumption that faster empirical antibiotic treatment is uniformly beneficial in all ICU pneumonia patients and underscore the importance of an individualized, context-dependent approach to antimicrobial therapy in critical care. While empirical antibiotics remain essential in the management of suspected infections, the results highlight potential risks of reflexively initiating treatment very early when diagnostic certainty is limited, particularly in the absence of shock or clear sepsis. The absence of a consistent survival signal with higher dosing frequency or more intensive antimicrobial regimens further suggests that escalation of therapy does not necessarily translate into improved outcomes and may reflect survivorship or confounding by indication rather than therapeutic benefit. Future prospective studies are needed to define the optimal timing, selection, and intensity of antibiotic therapy in well-characterized ICU pneumonia populations, ideally incorporating validated severity-of-illness metrics, microbiological data, and time-varying or causal-inference-oriented analytic approaches. Until such evidence becomes available, clinicians should balance the urgency of timely treatment with diagnostic precision and antimicrobial stewardship principles, avoiding automatic early use of broad-spectrum agents in situations of diagnostic uncertainty.

## Figures and Tables

**Table 1:** Descriptive statistics by timing group. Baseline characteristics and outcomes for early (≤3 h) versus delayed (>3 h) initiation of antibiotic therapy among patients admitted to the intensive care unit (ICU). Age and sex represent baseline characteristics, whereas ICU length of stay (LOS) and mortality are post-admission outcomes and may be influenced by survivorship and downstream clinical trajectories. Accordingly, ICU LOS is presented descriptively and should not be interpreted as a baseline measure or as evidence of causal effects.

Group	N	Mean Age (Years) ± SD	Median ICU LOS [min–max] (Days)	Mean ICU LOS (Days) ± SD	Mortality (%)
Early	4279	63.6 ± 15.5	3.4 [1–226.4]	6.7 ± 8.7	26.1
Delayed	3290	63.6 ± 15.9	4.0 [1–76.1]	7.4 ± 9.2	21.5

Abbreviations: ICU, intensive care unit; LOS, length of stay; SD, standard deviation.

**Table 2:** Gender distribution by timing group. Number and percentage of male and female patients according to antibiotic timing among patients admitted to the intensive care unit (ICU).

Group	Male, *n* (%)	Female, *n* (%)
Early (*n* = 4279)	2439 (57.0)	1840 (43.0)
Delayed (*n* = 3290)	1893 (57.5)	1397 (42.5)

**Table 3:** Most frequently prescribed antibiotics. List of the most commonly used antibiotics in the study cohort of patients admitted to the intensive care unit (ICU). Frequencies reflect overall prescribing patterns; patients may have received more than one antibiotic, and no causal or comparative inference regarding outcomes can be drawn from these counts.

Antibiotic	Frequency	Percentage (%)
Cefepime	2212	29.2
Ceftriaxone	1365	18.0
Piperacillin–tazobactam	988	13.1
Azithromycin	620	8.2
Cefazolin	510	6.7
Levofloxacin	329	4.3
Amoxicillin–clavulanic acid	309	4.1
Ceftazidime	245	3.2

**Table 4:** Multivariable logistic regression analysis of hospital mortality. Adjusted odds ratios (ORs) with 95% confidence intervals (CIs) for predictors of in-hospital mortality among patients admitted to the intensive care unit (ICU). The primary etiologic model adjusted for age and sex only. Additional terms shown are presented for exploratory, descriptive purposes and do not represent baseline confounder adjustment.

Variable	Odds Ratio (OR)	95% CI (Lower)	95% CI (Upper)	*p* Value
Intercept	0.08	0.06	0.10	**<0.0001**
Male sex (vs. female)	1.02	0.92	1.14	0.7247
Early antibiotic administration (≤3 h vs. >3 h)	1.30	1.16	1.44	**<0.0001**
Age (per 1-year increase)	1.02	1.02	1.02	**<0.0001**
ICU admission	0.07	0.05	0.09	**<0.0001**
ICU length of stay (per day, exploratory/downstream)	1.02	1.01	1.03	**<0.0001**

Abbreviations: CI, confidence interval; ICU, intensive care unit; LOS, length of stay; OR, odds ratio. ICU length of stay represents a post-admission, downstream variable that may be influenced by survival and clinical decision-making and should not be interpreted as a causal or baseline predictor of mortality. Note: Statistically significant *p* values are shown in bold.

**Table 5:** Intensive care unit length of stay and mortality by antibiotic and timing group. Comparison of early (≤3 h) versus delayed (>3 h) antibiotic administration with respect to intensive care unit length of stay (ICU LOS) and in-hospital mortality. Reported *p* values refer exclusively to comparisons of ICU LOS and not to mortality. Mortality proportions are presented descriptively only and should not be interpreted as evidence of antibiotic-specific effects. Differences are subject to strong confounding by indication, survivorship bias, and substantial group-size imbalances.

Antibiotic	*n* (Early)	*n* (Delayed)	Mortality, Early (%)	Mortality, Delayed (%)	*p* Value (ICU LOS)
Levofloxacin	176	153	21.6	13.1	0.3400
Amoxicillin–clavulanic acid	21	24	28.6	4.2	0.1686
Piperacillin–tazobactam	556	432	25.5	23.4	**0.0014**
Cefepime	1191	1021	28.2	27.6	**<0.0001**
Ceftriaxone	761	604	23.3	19.9	0.1257
Ceftazidime	115	130	25.2	23.1	0.2349
Azithromycin	329	291	25.2	9.3	0.2547
Cefazolin	284	226	20.4	16.8	**0.0001**

Abbreviations: ICU, intensive care unit; LOS, length of stay. *p* values refer to comparisons of ICU LOS between early and delayed groups. Mortality percentages are presented descriptively and were not subjected to statistical testing. Note: Statistically significant *p* values are shown in bold.

**Table 6:** Outcomes by number of different antibiotics administered. Summary of mortality, age, and intensive care unit length of stay (ICU LOS) according to the number of unique antibiotics administered per patient. Statistical comparisons were performed using the chi-square test for mortality (1 antibiotic vs. ≥2 antibiotics; *p* < 0.001) and Welch’s *t*-test for ICU length of stay (1 antibiotic vs. ≥2 antibiotics; *p* = 0.130). Because patients must survive long enough to receive multiple antibiotics, comparisons across antibiotic-count categories are subject to substantial survivorship and indication bias and should be interpreted descriptively.

Number of Antibiotics	N	Mean Age (± SD)	Median ICU LOS (Days)	Mean ICU LOS (± SD)	Mortality (%)
1	4952	64.3 ± 16.1	3.63	6.8 ± 9.2	24.7
2	251	62.5 ± 13.8	2.96	5.8 ± 8.5	10.0
3	38	58.2 ± 14.3	2.88	6.7 ± 11.0	10.5
≥4	3	70.0 ± 10.8	16.83	14.0 ± 10.9	0.0

Abbreviations: ICU, intensive care unit; LOS, length of stay; SD, standard deviation. Higher antibiotic counts reflect downstream treatment intensity and do not represent baseline exposure groups.

**Table 7:** Odds ratios for in-hospital mortality by antibiotic (≥3 vs. <3 doses). Association between the number of antibiotic doses (≥3 versus <3 doses) and in-hospital mortality for the most frequently prescribed antibiotics in the study cohort of patients admitted to the intensive care unit (ICU). For each antibiotic, the number of patients receiving fewer than three doses versus three or more doses is reported, together with the odds ratio (OR), 95% confidence interval (CI), and *p* value derived from univariable logistic regression. Because patients must survive long enough to receive multiple doses, these analyses are subject to substantial survivorship and indication bias and should be interpreted as exploratory and hypothesis-generating only. An OR < 1 indicates lower mortality associated with higher dosing frequency, whereas an OR > 1 indicates higher mortality.

Antibiotic	*n* < 3 Doses	*n* ≥ 3 Doses	Odds Ratio (OR)	95% CI (Lower)	95% CI (Upper)	*p* Value
Cefepime	1479	117	0.92	0.60	1.41	0.7053
Ceftriaxone	981	56	1.24	0.66	2.31	0.5012
Azithromycin	707	36	1.58	0.73	3.45	0.2481
Piperacillin–tazobactam	650	50	0.99	0.51	1.94	0.9805
Cefazolin	329	29	1.86	0.78	4.40	0.1600
Levofloxacin	259	9	4.38	1.13	17.02	**0.0329**
Ceftazidime	191	5	2.04	0.33	12.60	0.4416
Amoxicillin–clavulanic acid	31	3	4.67	0.32	68.03	0.2598
Cefpodoxime proxetil	11	1	Not estimable	-	-	-

Abbreviations: CI, confidence interval; OR, odds ratio. Odds ratios for cefpodoxime proxetil could not be reliably estimated due to sparse data (*n* = 12). Dose-based comparisons reflect downstream treatment exposure and do not represent baseline risk groups. Note: Statistically significant *p* values are shown in bold.

## Data Availability

The dataset analyzed in this study is available through the MIMIC-IV database hosted on PhysioNet (https://physionet.org/content/mimiciv/2.2/) and can be accessed upon completion of the required credentialing and data use agreement process (accessed on 15 September 2025).
